# Early Tumor Shrinkage as a Predictive Factor for Outcomes in Hepatocellular Carcinoma Patients Treated with Lenvatinib: A Multicenter Analysis

**DOI:** 10.3390/cancers12030754

**Published:** 2020-03-23

**Authors:** Aya Takahashi, Michihisa Moriguchi, Yuya Seko, Toshihide Shima, Yasuhide Mitsumoto, Hidetaka Takashima, Hiroyuki Kimura, Hideki Fujii, Hiroki Ishikawa, Takaharu Yo, Hiroshi Ishiba, Atsuhiro Morita, Masayasu Jo, Yasuyuki Nagao, Masahiro Arai, Tasuku Hara, Akira Okajima, Akira Muramatsu, Naomi Yoshinami, Tomoki Nakajima, Hironori Mitsuyoshi, Atsushi Umemura, Taichiro Nishikawa, Kanji Yamaguchi, Takeshi Okanoue, Yoshito Itoh

**Affiliations:** 1 Department of Gastroenterology and Hepatology, Kyoto Prefectural University of Medicine, Kyoto 602-8566, Japan; 2 Department of Gastroenterology and Hepatology, Saiseikai Suita Hospital, Suita 564-0013, Japan; 3 Department of Gastroenterology, Osaka General Hospital of West Japan Railway Company, Osaka 545-0053, Japan; 4 Department of Gastroenterology, Japanese Red Cross Kyoto Daiichi Hospital, Kyoto 605-0981, Japan; 5 Department of Gastroenterology and Hepatology, Omihachiman Community Medical Center, Omihachiman 523-0082, Japan; 6 Department of Gastroenterology and Hepatology, North Medical Center of Kyoto Prefectural University of Medicine, Yosagun 629-2261, Japan; 7 Department of Gastroenterology, Japanese Red Cross Kyoto Daini Hospital, Kyoto 602-8026, Japan; 8 Department of Gastroenterology, Otsu City Hospital, Otsu 520-0804, Japan; 9 Department of Gastroenterology, Matsushita Memorial Hospital, Moriguchi 570-8540, Japan; 10 Department of Gastroenterology, Kyoto Yamashiro General Medical Center, Kizugawa 619-0214, Japan; 11 Department of Gastroenterology, Fukuchiyama City Hospital, Fukuchiyama 620-8505, Japan; 12 Department of Gastroenterology, Koseikai Takeda Hospital, Kyoto 600-8558, Japan; 13 Department of Gastroenterology, Akashi City Hospital, Akashi 673-8501, Japan; 14 Department of Gastroenterology, Kyoto City Hospital, Kyoto 604-8845, Japan; 15 Department of Gastroenterology, Saiseikai Kyoto Hospital, Kyoto 617-0814, Japan; 16 Department of Gastroenterology and Hepatology, Kyoto Chubu Medical Center, Kyoto 629-0197, Japan

**Keywords:** hepatocellular carcinoma, lenvatinib, early tumor shrinkage, overall survival, Response Evaluation Criteria in Solid Tumors (RECIST)

## Abstract

We investigated the association between early tumor shrinkage (ETS) and treatment outcome in patients with hepatocellular carcinoma treated with lenvatinib (LEN). A retrospective analysis was performed in 104 patients. ETS was defined as tumor shrinkage at the first evaluation in the sum of target lesions’ longest diameters from baseline according to the Response Evaluation Criteria in Solid Tumors (RECIST). The median overall survival (OS) was not reached, whereas the median progression-free survival (PFS) was 5.0 months. The receiver operating characteristic curve analysis in differentiating long-term responders (PFS ≥ 5.0 months) from short-term responders (PFS < 5.0 months) revealed an ETS cut-off value of 10%. ETS ≥ 10% was significantly correlated with better PFS and OS compared with ETS < 10%. Additionally, ETS ≥ 10% showed a better discrimination ability on prognosis compared with modified RECIST-based objective response at the first evaluation. Multivariate analysis confirmed ETS ≥ 10% as an independent predictor of better OS, as well as a Child–Pugh score of 5 and macrovascular invasion. In conclusion, ETS ≥ 10% was strongly associated with outcome in patients treated with LEN. This biomarker could allow earlier assessment of the treatment response and guide treatment decision-making for HCC.

## 1. Introduction

Hepatocellular carcinoma (HCC) is the sixth most common malignancy and the fourth leading cause of cancer-related death worldwide [1]. Because the symptoms of early HCC are often inconspicuous, most patients are diagnosed at an advanced stage, eliminating the option of local treatment, such as curative hepatic resection, tumor ablation, or transarterial therapy. Therefore, systemic treatment of advanced HCC is of great concern.

Sorafenib, a multikinase inhibitor, is the first targeted agent approved as first-line therapy for advanced HCC [2]. In the last two years, three successful novel drugs have emerged from clinical trials for clinical use, including lenvatinib (LEN) as a first-line treatment and regorafenib and ramucirumab as second-line treatments [3,4,5]. With the increasing number of therapeutic options, the need for effective early methods to evaluate treatment activity has become critical. 

Early tumor shrinkage (ETS) is defined as a reduction in tumor size at the first radiologic evaluation (i.e., at eight weeks after treatment initiation) [6]. A correlation between ETS and treatment outcome has been reported in many malignancies such as metastatic colorectal cancer [6,7,8], pancreatic cancer [9], renal cell carcinoma [10], and non-small cell lung cancer [11]. The benefit of utilizing ETS as a marker is that it might enable earlier prediction of drug activity compared with conventional end points such as progression-free survival (PFS) and overall survival (OS). This association, however, has not been discussed in HCC patients undergoing systemic therapy owing to an inadequate response rate to sorafenib treatment.

In the phase 3 REFLECT trial, LEN demonstrated a significantly higher response rate than did sorafenib (18.8% vs. 6.5% according to the Response Evaluation Criteria in Solid Tumors (RECIST) and 40.6% vs. 12.4% according to the modified RECIST (mRECIST)) [12,13]. Recently, a post-hoc analysis of the REFLECT trial reported that the objective response based on the mRECIST was an independent predictor of OS [14]. In addition, we previously reported that 95.5% of responders achieved an objective response at the first evaluation after LEN initiation [15]. Therefore, we hypothesized that ETS may also reflect the treatment outcome of patients with HCC. This study aimed to evaluate the prognostic role of ETS, as well as identify the optimal cut-off value of ETS in HCC patients treated with LEN. We adopted the RECIST criteria to evaluate treatment response, corresponding with previous studies on ETS [6,7,8,9,10,11].

## 2. Results

### 2.1. Patient Characteristics

Among 146 patients treated with LEN, we enrolled 104 (71.2%) patients in the present study. The clinical characteristics of the 104 study patients are summarized in Table 1. The median age of the patients was 74 years, and 74.0% were men. Most patients had an Eastern Cooperative Oncology Group performance status (ECOG-PS) of 0/1 (96.2%) and Child–Pugh A (91.3%), and 3, 38, and 63 patients had Barcelona Clinic Liver Cancer (BCLC) stage A, B, and C, respectively.

The median follow-up period was 11.0 months; the median PFS and post-progression survival (PPS) were 5.0 and 8.5 months, whereas the median OS was not reached.

### 2.2. Definition of Early Tumor Shrinkage

**Figure 1:**
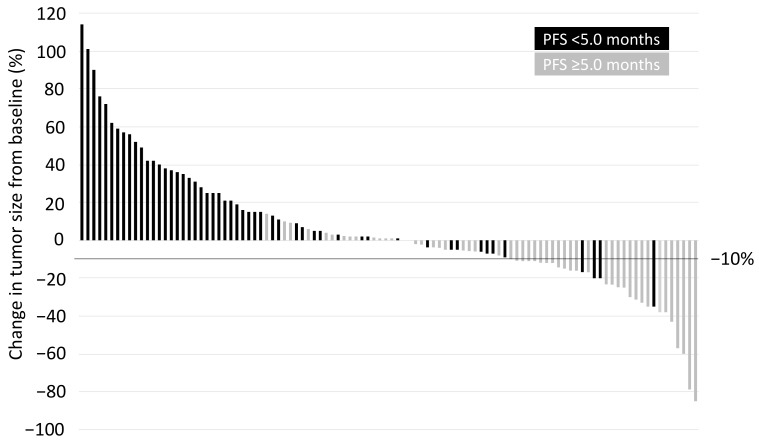
Waterfall plot of early tumor shrinkage based on the RECIST at the first evaluation. RECIST, response evaluation criteria in solid tumors; PFS, progression-free survival.

All patients had one or more measurable lesions based on the RECIST criteria. Figure 1 shows a waterfall plot of the ETS. The median change in ETS was +1.0%, with a range of −85% to +114%. Of the 104 patients, 54 (51.9%) and 50 (48.1%) were classified in the long-term (PFS ≥ 5.0 months) and short-term (PFS < 5.0 months) responders, respectively.

**Figure 2:**
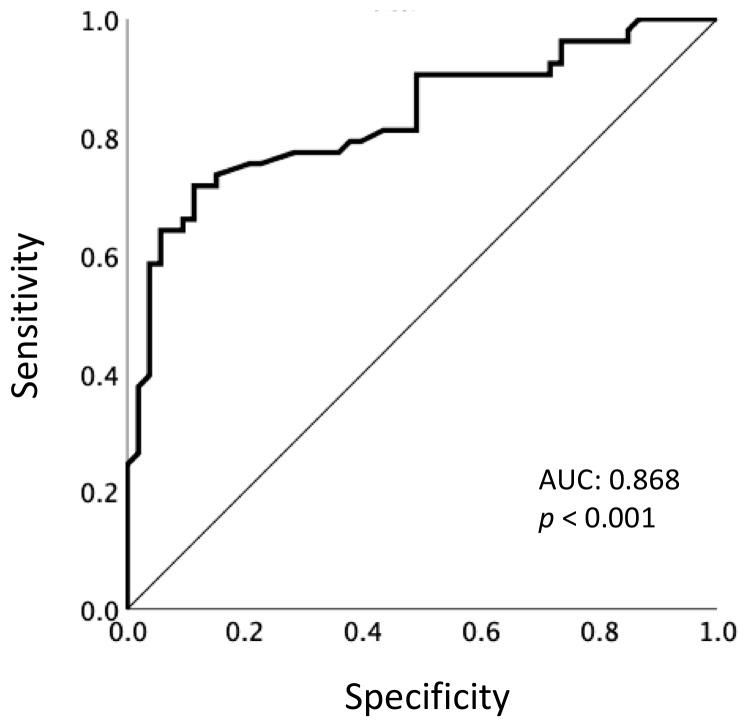
Receiver operating characteristic curve of the association between early tumor shrinkage and long-term response (PFS ≥ 5.0 months). PFS, progression-free survival; AUC, area under the curve.

Figure 2 shows the results of the receiver operating characteristic (ROC) analysis performed to determine the ability of ETS to predict PFS (<5.0 vs. ≥5.0 months). The area under the ROC curve was 0.868, and the optimal cut-off value of ETS was identified as 10%, which yielded a sensitivity of 80.0% and specificity of 74.1%. Additionally, when ETS ≥ 30% (i.e., the cut-off value for objective response) was used, the specificity was considerably worse, at 20.4%, although the sensitivity increased to 98.0%.

On the basis of these results, we determined that a 10% ETS best predicted PFS. Among the 104 patients, ETS ≥ 10% was achieved in 32 (30.8%) patients, and the baseline clinical profiles of patients with ETS ≥ 10% compared with ETS < 10% are shown in Table 1. Patients with ETS ≥ 10% were more likely to have favorable Child–Pugh score (*p* = 0.055), fewer than five tumors (*p* = 0.062), and small maximum diameter of lesions (*p* = 0.012).

### 2.3. Correlation of Early Tumor Shrinkage with AFP Response and Relative Dose Intensity

The median α-fetoprotein (AFP) ratio at eight weeks and relative dose intensity (RDI) at eight weeks were 0.91% (0.01–24.2%) and 71.0% (8.2–100%), respectively. We also performed the ROC analysis to determine the optimal cut-off values of AFP ratio and RDI at 8 weeks to predict PFS (<5.0 vs. ≥5.0 months). The area under the ROC curves of the AFP ratio and RDI at eight weeks were 0.600 and 0.644, and the optimal cut-off values were identified as 1.1 and 70%, respectively (Figure S1). ETS ≥ 10% was significantly associated with AFP ratio at eight weeks < 1.1 (*p* = 0.028) (Table 2), although there was no correlation between ETS and baseline AFP level (Table 1). Moreover, patients with ETS ≥ 10% had significant correlation with RDI at eight weeks ≥ 70% (*p* = 0.010) (Table 2).

### 2.4. Treatment Outcome Based on Early Tumor Shrinkage

**Figure 3:**
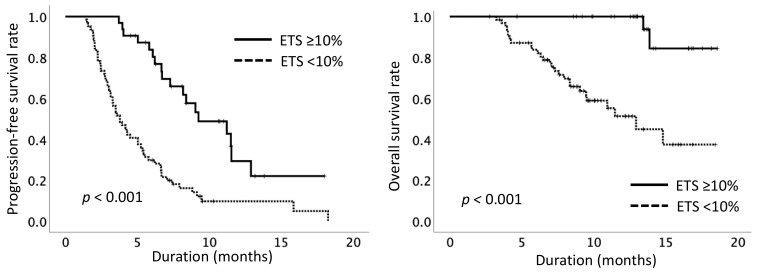
Cumulative progression-free survival and overall survival rates according to early tumor shrinkage. ETS, early tumor shrinkage.

Patients with ETS ≥ 10% had a longer PFS than that of patients with ETS < 10% (median 9.3 vs. 3.3 months, *p* < 0.001) (Figure 3).

Factors associated with a significant improvement in PFS included ETS ≥ 10%, Child–Pugh score of 5, baseline AFP level < 200 ng/mL, AFP ratio at eight weeks < 1.1, and RDI at eight weeks ≥ 70%. In the multivariate analysis, ETS ≥ 10% was significantly associated with improved PFS in comparison with ETS < 10% (hazard ratio (HR), 0.275 (0.157–0.483), *p* < 0.001). Another independent variable associated with improving PFS was baseline AFP level < 200 ng/mL (Table 3).

Next, we analyzed the impact of ETS on PPS and OS. Patients with ETS ≥ 10% experienced better PPS than patients with ETS < 10% (median not reached vs. 6.9 months, *p* = 0.002). Thus, we also investigated the rate of subsequent therapies after LEN and found that the patients achieving ETS ≥ 10% were more likely than those with ETS < 10% to receive subsequent therapies (84.2% vs. 49.0%, *p* = 0.013), including transarterial chemoembolization and other targeted therapies. In addition to a Child–Pugh score of 5, number of lesions < 5, absence of macrovascular invasion (MVI), and RDI at eight weeks ≥ 70%, ETS ≥ 10% was a significant predictive factor of OS in the univariate analysis (ETS ≥ 10% vs. ETS < 10%: median not reached vs. 12.3 months, *p* < 0.001) (Table 4). Thus, the factors ETS, Child–Pugh score, number of lesions, MVI, and RDI at eight weeks were entered into the multivariate analysis, which confirmed that ETS ≥ 10% (HR, 0.091 (0.021–0.392), *p* = 0.001), a Child–Pugh score of 5 (HR, 0.424 (0.199–0.902), *p* = 0.026), and absence of MVI (HR, 0.454 (0.222–0.926), *p* = 0.030) were independent prognostic factors (Table 4). Additionally, the impact of ETS on OS was consistent across subgroups based on Child–Pugh score and MVI (Figure S2).

### 2.5. Comparison of Early Tumor Shrinkage with Modified RECIST/RECIST-Based Objective Response

At the first evaluation, there were 12 (15.0%) objective responses based on the RECIST and 35 (33.7%) based on the mRECIST. The objective response based on both the mRECIST and RECIST at the first evaluation was correlated with ETS ≥ 10% (Table 5), and also significantly associated with OS in the univariate analysis (mRECIST-based objective response vs. non-objective response: median not reached vs. 13.4 months, *p* = 0.004; RECIST-based objective response vs. non-objective response: median not reached vs. 13.9 months, *p* = 0.006).

Next, we compared the discrimination abilities of ETS with mRECIST/RECIST-based objective response at the first evaluation on prognosis. ETS ≥ 10% showed a better discrimination ability on prognosis compared with mRECIST/RECIST-based objective response at the first evaluation (c-index: ETS, 0.69 (0.64–0.74); mRECIST, 0.62 (0.56–0.69); RECIST, 0.58 (0.54–0.62)) (Table 6).

## 3. Discussion

The present analysis is one of the first to investigate the role of ETS in HCC patients treated with LEN. On the basis of our results, ETS ≥ 10% was significantly associated with survival outcome. As no predictive biomarker of treatment efficacy has been established in HCC patients undergoing systemic therapy [16,17], this early indicator of LEN sensitivity is a potential predictive factor that can easily be applied in clinical settings. To the best of our knowledge, our report is the first to demonstrate the prognostic value of ETS in HCC patients treated with LEN.

Here, we clearly identified an ETS value of 10% as the optimal cut-off for predicting a long-term response to LEN (PFS ≥ 5.0 months), based on ROC analysis. This finding is in line with previous reports of various malignancies treated with chemotherapy including targeted therapies, using a range of ETS cut-off values (10%–30%) to determine predictors of PFS and OS [6,7,8,9,10,11]. Additionally, when we used the standard cut-off value for objective response, that is, ETS ≥ 30%, the specificity for predicting PFS was considerably worse, compared with ETS ≥ 10%, because only 15% of the patients achieved that ETS. In the present study, median PFS was 5.0 months, which was shorter than that in the phase 3 REFLECT trial, revealing median PFS was 7.4 months in the overall population and 7.2 months in the Japanese subset [18]. However, in real-world settings, median PFS ranging from 4.4 to 5.4 months has been reported, consistent with our results [19,20]. This may be explained by more patients in real-world settings being aged and having low ECOG-PS score and Child–Pugh score compared with the clinical trial. Thus, we consider that using a cut-off of PFS ≥ 5.0 months was acceptable in the clinical practice.

We additionally analyzed the impact of ETS on survival outcomes. Our multivariate analysis confirmed that ETS ≥ 10% was an independent predictor of an improved OS, together with well-known clinical prognostic variables in HCC patients, such as favorable liver function and MVI [16,21]. It is acceptable that tumor shrinkage after a short treatment period reflects treatment sensitivity and correlates with the response duration; however, it is not clear why ETS markedly affected patient prognosis. Therefore, we clarified that ETS was significantly correlated with not only PFS, but also PPS. One of the reasons for the association between ETS and PPS might be that patients who achieve greater ETS (≥10 vs. <10%) by the first evaluation have a reduced tumor burden and prolong the time to lethal tumor load, thereby increasing their chance of receiving subsequent therapies, even after disease progression (PD) (*p* = 0.013). This may explain why ETS showed a strong influence on patient prognosis in the present study. Similarly, in a post-hoc analysis in a phase 3 trial of colon cancer, Mazard et al. revealed that achieving greater tumor shrinkage at the nadir compared with baseline was correlated with a longer PFS, as well as PPS [22]. In HCC patients treated with sorafenib, PPS showed a strong correlation with OS [23] and was related to the patients’ baseline characteristics, such as ECOG-PS and tumor factors, as well as time-to-progression and PD patterns [24,25]. In patients treated with LEN, which resulted in a higher response rate compared with sorafenib, the degree of treatment response might be predictive of PPS. However, because patients with ETS ≥ 10% had favorable baseline liver function and smaller size and number of lesions in the present study, these factors could also influence PPS. Thus, further studies are needed to confirm the relationship between PPS and the maximum tumor reduction during LEN treatment.

According to a post-hoc analysis of the phase 3 REFLECT trial, the objective response based on mRECIST was an independent predictor of OS [14]. In addition, the correlation between the mRECIST-based objective response and OS has been demonstrated with other targeted therapies (e.g., brivanib, nintedanib, and sorafenib) according to data from prospective randomized trials on HCC [26,27]. Therefore, we compared the discrimination abilities of ETS and mRECIST-based objective response at the first evaluation on prognosis. As a result, the c-index of ETS was superior to that of the mRECIST-based objective response. Originally, the mRECIST criteria was derived from the concept that tumor necrosis was induced by treatment. With LEN therapy, however, we have sometimes experienced that tumor growth occurs despite disappearance of arterial tumor enhancement, or that arterial tumor enhancement reappears soon after LEN interruption [28]. Kuzuya et al. indicated that a good radiologic antitumor response, based on the mRECIST (i.e., almost complete disappearance of arterial tumor enhancement), might not necessarily imply tumor necrosis, especially soon after LEN initiation [29]. Thus, it is sometimes difficult to clearly distinguish between viable tumor tissue and necrotic tissue during the early phase of treatment. On the other hand, the RECIST-based measurement of tumor size reduction may certainly reflect antitumor activity, regardless of the evaluation time points. This could be a potential explanation for the results of our study. Moreover, the RECIST-based measurement of tumor size may be simpler and have less interobserver variation compared with the mRECIST. For these reasons, we considered that ETS ≥ 10% based on the RECIST could be a more favorable predictive factor than the mRECIST-based objective response at the first evaluation in terms of early indicator of survival outcome.

In the present study, 30.8% of patients achieved ETS ≥ 10%. We found that patients with a favorable Child–Pugh score, fewer tumor numbers, and small maximum diameter of lesions were more likely to achieve ETS ≥ 10%. Ueshima et al. reported that a baseline Child–Pugh score of 5 and albumin-bilirubin (ALBI) grade of 1 were factors predicting an objective response in HCC patients treated with LEN [30], supporting our results. Thus, we considered that favorable hepatic function was also important for achieving ETS. In addition to ETS, we investigated the other post-treatment factors such as AFP ratio and RDI at eight weeks and found that these factors were significantly correlated with ETS. This finding is consistent with a previous report, demonstrating the association of AFP response and imaging response in HCC patients treated with LEN [31]. RDI at eight weeks was reported to associate with not only imaging response, but also OS [15,32]. In the present study, RDI at eight weeks was an independent contributing factor for ETS (Table S1) and was significantly associated with PFS and OS in the univariate analysis. We believe that maintaining high RDI during the initial eight weeks is important for achieving ETS and prolonging patient’s outcomes.

Some patients revealed long survival without ETS ≥ 10% in our study. There are several common prognostic factors in HCC patients, regardless of treatment efficacy, such as favorable liver function, ECOG-PS, and tumor stage. According to a previous report of sorafenib treatment, no difference in OS was detected between the long stable disease group (i.e., ≥3 months) and objective response group [33]. Thus, objective response may not always be required for improving OS in sorafenib treatment. However, in LEN therapy, we consider that tumor shrinkage further improves patients’ survival, according to our results.

Recently, the modified ALBI (mALBI) grade, which divided grade 2 into two subgroups based on a cut-off of ALBI score −2.27 for predicting ICG-R 15 30%, has been proposed [34]; mALBI 2b or greater was shown to be a predictive factor for poor prognosis in LEN treatment [21]. In this study, mALBI of 1/2a had a tendency to predict better OS in univariate analysis (*p* = 0.089), but it was not an independent prognostic factor in multivariate analysis. This was probably because only 10% of Child–Pugh scores of 5 were classified as mALBI of 2b, but 14.5% of mALBI grade 1/2a were classified into Child–Pugh scores of 6 in our cohort.

Some limitations of our study should be acknowledged, including its retrospective nature, the limited number of patients, and the lack of detailed information on subsequent therapies. Additionally, we used median PFS for the determination of the ETS cut-off value, because OS did not reach median. We believe that the cut-off value determined by OS will be more desirable. In order to confirm the significance of ETS on prognosis, further validation studies in larger independent cohorts are definitely needed. Nevertheless, our study shows, for the first time, a direct correlation between ETS and outcomes in HCC patients treated with LEN.

## 4. Materials and Methods 

### 4.1. Study Design and Patients

This was an observational, retrospective, multicenter study on patients with unresectable HCC treated with LEN in routine clinical practice. From March 2018 to November 2019, 146 Japanese patients were treated with LEN at 16 institutions in Japan (Kyoto Prefectural University of Medicine (*n* = 35), Saiseikai Suita Hospital (*n* = 19), Osaka General Hospital of West Japan Railway Company (*n* = 17), Japanese Red Cross Kyoto Daiichi Hospital (*n* = 16), Omihachiman Community Medical Center (*n* = 15), North Medical Center of Kyoto Prefectural University of Medicine (*n* = 10), Japanese Red Cross Kyoto Daini Hospital (*n* = 8), Otsu City Hospital (*n* = 7), Matsushita Memorial Hospital (*n* = 4), Kyoto Yamashiro General Medical Center (*n* = 3), Fukuchiyama City Hospital (*n* = 3), Koseikai Takeda Hospital (*n* = 3), Akashi City Hospital (*n* = 2), Kyoto City Hospital (*n* = 2), Saiseikai Kyoto Hospital (*n* = 1), and Kyoto Chubu Medical Center (*n* = 1)). Of these patients, 104 patients were enrolled and divided into two groups, long-term responders (PFS ≥ median) and short-term responders (PFS < median) based on the RECIST. The exclusion criterion was those who were not evaluated for radiologic antitumor response at 6–8 weeks after LEN start for any reason. Data were obtained from clinical medical records, using a cut-off date of December 2019. The ethics committees of all facilities that participated in this study approved the present study protocol, which complied with all provisions of the declaration of Helsinki. 

### 4.2. Diagnosis and Treatment

HCC was diagnosed as described previously [35]. Each patient received LEN orally at 8 mg/day (body weight < 60 kg) or 12 mg/day (body weight ≥ 60 kg), although a lower starting dose was used in some patients according to the physician’s decision. LEN was administered until PD, unacceptable toxicity, or the patient’s decision to withdraw. Dose adjustments due to adverse events were performed according to routine clinical practice.

### 4.3. Assessments

Tumor assessment was carried out at baseline and every 6–8 weeks until evidence of PD on enhanced computed tomography (CT) and/or magnetic resonance imaging (MRI). Treatment activity was evaluated according to the RECIST (version 1.0) guidelines by two hepatic physicians at Kyoto Prefectural University of Medicine. ETS was defined as a relative change in the sum of longest diameters of RECIST target lesions at the first evaluation (6–8 weeks after starting LEN) compared with baseline, consistent with previous studies on ETS [6,7,8,9,10,11]. RECIST target lesions were all measurable lesions up to a maximum of five lesions per organ and 10 lesions in total. Unmeasurable lesions such as MVI were not included in the evaluation of ETS. Each patient was also classified as objective response (complete response/partial response) or non-objective response (stable disease/PD) at the first evaluation, according to the RECIST/mRECIST guidelines. Additionally, we investigated the other post-treatment factors, including AFP response and RDI of LEN at eight weeks. The concentration of serum AFP was measured once a month after the start of LEN treatment. The AFP ratio at eight weeks was calculated as AFP value at eight weeks/baseline AFP value. RDI at eight weeks was defined as the actual dose delivered during the initial eight weeks/standard dose (body weight ≥ 60 kg: 12 mg × 8 weeks; < 60 kg: 8 mg × 8 weeks).

### 4.4. Statistical Analysis

ROC analysis was performed to identify the optimal cutoff value for ETS, discriminating the long-term responders (PFS ≥ median) from the short-term responders (PFS < median). Using the ETS cut-off value obtained by ROC analysis, the associations of ETS with various clinical parameters, including age, gender, ECOG-PS, body weight, Child–Pugh score, etiology, maximum diameters of lesions, number of lesions, EHS, MVI, BCLC stage, AFP level, prior history of systemic therapy, AFP ratio at eight weeks, and RDI at eight weeks, were investigated. The cut-off values of maximum diameters and number of lesions were determined based on median values. Univariate analyses were performed using Fisher’s exact test and Mann–Whitney U-test, as appropriate. The prognostic impacts of ETS on PFS, PPS, and OS were evaluated. PPS was calculated from the date of PD to death from any cause or the last follow-up. The distributions of PFS, PPS, and OS were estimated using the Kaplan–Meier method and compared with the log-rank test. Predictive discrimination ability on prognosis was estimated by c-index [36]. Hazard ratios (HRs) with 95% confidence intervals were calculated via multivariate analyses using the Cox hazards model. A *p*-value < 0.05 was considered statistically significant. Statistical analyses were conducted using SPSS software ver. 25 (SPSS, Chicago, IL, USA) and SAS 9.4 (SAS Institute, Cary, NC, USA).

## 5. Conclusions

We found that ETS was strongly associated with the prognosis of HCC patients treated with LEN. Achieving rapid tumor shrinkage consistently delays tumor progression and prolongs survival, thus enabling earlie assessment of the treatment outcome and guiding treatment decision-making for HCC. Additional prospective studies are needed to evaluate the role of ETS as a surrogate marker of prognosis.

## Figures and Tables

**Table 1:** Baseline patient characteristics, according to early tumor shrinkage (*n* = 104) ^†^.

Variable	Total (*n* = 104)	ETS ≥ 10% (*n* = 32)	ETS < 10% (*n* = 72)	*p* ^‡^
Age, years (range)	74 (48–93)	71 (53–91)	75 (48–93)	0.510
Sex, male/female	77/27	26/6	51/21	0.336
ECOG PS, 0/1/2	80/20/4	26/5/1	54/15/3	0.831
Body weight, kg (range)	57.7 (38.0–103.0)	58.8 (41.5–90.0)	57.3 (38.0–103.0)	0.938
Etiology, HCV/HBV/alcohol/others	40/17/20/27	14/5/5/8	26/12/15/19	0.294
Child–Pugh score, 5/6/7	59/36/9	23/6/3	36/30/6	**0.055**
Maximum diameter of lesions, mm	34.5 (10–125)	28.0 (10–124)	37.3 (10–125)	**0.012**
Number of lesions, <5/≥5	54/50	21/11	33/39	**0.062**
EHS, +/−	42/62	13/19	29/43	1.000
MVI, +/−	18/86	4/28	14/58	0.575
BCLC stage, A/B/C	3/38/63	1/12/19	2/26/44	0.984
AFP, ng/mL (range)	94.3 (1.2–185772)	113.2 (2.8–25881)	92.5 (1.2–185772)	0.404
1st/2nd/3rd line	72/19/13	23/7/2	49/12/11	0.403
Initial dose of LEN, full dose/reduced dose	85/19	29/3	56/16	0.171

Abbreviations: ETS, early tumor shrinkage; ECOG, Eastern Cooperative Oncology Group; PS, performance status; HCV, hepatitis C virus; HBV, hepatitis B virus; EHS, extrahepatic spread; MVI, macrovascular invasion; BCLC, Barcelona Clinic Liver Cancer; AFP, alpha-fetoprotein; LEN, lenvatinib. ^†^ Results are presented as numbers for qualitative data or as medians for quantitative data. ^‡^ Bold font for *p*-values indicates less than 0.1.

**Table 2:** Correlation of early tumor shrinkage with AFP response and relative dose intensity ^†^.

Variable	ETS ≥ 10% (*n* = 32)	ETS < 10% (*n* = 72)	*p* ^‡^
AFP ratio at 8 weeks, <1.1/≥1.1	25/7	39/33	**0.028**
RDI at 8 weeks, ≥70%/<70%	23/9	31/42	**0.010**

Abbreviations are defined in Table 1. RDI, relative dose intensity. ^†^ Results are presented as numbers. ^‡^ Bold font for *p*-values indicates less than 0.05.

**Table 3:** Univariate and multivariate analyses for progression-free survival.

Factors	Univariate Analysis			Multivariate Analysis ^†^		
	HR	95% CI	*p* ^‡^	HR	95% CI	*p* ^‡^
Age, <75 years	0.688	0.440–1.076	0.102			
Gender, male	0.635	0.390–1.034	0.068			
ECOG-PS, 0	0.721	0.433–1.203	0.211			
Child-Pugh score, 5	0.617	0.398–0.958	**0.031**	0.885	0.556–1.410	0.608
Maximum diameter of lesions, <35 mm	0.780	0.506–1.201	0.259			
Number of lesions, <5	0.650	0.420–1.003	0.052			
EHS, absence	0.841	0.541–1.307	0.441			
MVI, absence	0.842	0.481–1.475	0.547			
AFP, <200 ng/mL	0.552	0.357–0.855	**0.008**	0.476	0.299–0.759	**0.004**
AFP ratio at 8 weeks, <1.1	0.641	0.412–0.997	**0.049**	0.912	0.569–1.462	0.702
RDI at 8 weeks, ≥70%	0.618	0.401–0.953	**0.030**	0.763	0.484–1.205	0.246
ETS, ≥10%	0.275	0.161–0.469	**<0.001**	0.275	0.157–0.483	**<0.001**

Abbreviations are defined in Table 1 and Table 2. HR, hazard ratio; CI, confidence interval. ^†^ Estimated using Cox regression analysis. ^‡^ Bold font for *p*-values indicates less than 0.05.

**Table 4:** Univariate and multivariate analyses for overall survival.

Factors	Univariate Analysis			Multivariate Analysis ^†^		
	HR	95% CI	*p* ^‡^	HR	95% CI	*p* ^‡^
Age, <75 years	0.598	0.305–1.169	0.133			
Gender, male	0.575	0.286–1.158	0.122			
ECOG-PS, 0	0.623	0.304–1.274	0.195			
Child-Pugh score, 5	0.403	0.205–0.794	**0.009**	0.424	0.199–0.902	**0.026**
Maximum diameter of lesions, <35 mm	0.593	0.304–1.156	0.125			
Number of lesions, <5	0.443	0.220–0.891	**0.022**	0.474	0.222–1.014	0.054
EHS, absence	0.880	0.450–1.722	0.710			
MVI, absence	0.455	0.211–0.981	**0.044**	0.454	0.222–0.926	**0.030**
AFP, <200 ng/mL	0.593	0.305–1.155	0.125			
AFP ratio at 8 weeks, <1.1	0.733	0.377–1.425	0.359			
RDI at 8 weeks, ≥70%	0.429	0.252–0.957	**0.037**	0.787	0.379–1.634	0.521
ETS, ≥10%	0.090	0.020–0.394	**<0.001**	0.091	0.021–0.392	**0.001**

Abbreviations are defined in Table 1, Table 2 and Table 3. ^†^ Estimated using Cox regression analysis. ^‡^ Bold font for *p*-values indicates less than 0.05.

**Table 5:** The association between early tumor shrinkage and mRECIST-/RECIST-based objective response at the first evaluation ^†^.

Variable	ETS ≥ 10% (*n* = 32)	ETS < 10% (*n* = 72)	*p* ^‡^
mRECIST, objective response/non-objective response	22/10	13/59	**<0.001**
RECIST, objective response/non-objective response	12/20	0/72	**<0.001**

Abbreviations are defined in Table 1. mRECIST, modified Response Evaluation Criteria in Solid Tumors; RECIST, Response Evaluation Criteria in Solid Tumors. ^†^ Results are presented as numbers. ^‡^ Bold font for *p*-values indicates less than 0.05.

**Table 6:** The discrimination abilities on prognosis.

Variable	C-Index (95% CI)
ETS, ≥ 10/< 10 %	0.69 (0.64–0.74)
mRECIST, objective response/non-objective response ^†^	0.62 (0.56–0.69)
RECIST, objective response/non-objective response ^†^	0.58 (0.54–0.62)

Abbreviations are defined in Table 4 and Table 5. ^†^ Evaluated at the first evaluation.

## Supplementary Materials

The following are available online at https://www.mdpi.com/2072-6694/12/3/754/s1, Figure S1: Receiver operating characteristic curves of the association of long-term response (PFS ≥ 5.0 months) with AFP ratio (A) and RDI (B) at 8 weeks, Figure S2: Subgroup analyses for overall survival. ETS, early tumor shrinkage; CPS, Child-Pugh score; MVI, macrovascular invasion, Table S1: Factors associated with ETS ≥ 10%.

## References

[1] Llovet J.M., Zucman-Rossi J., Pikarsky E., Sangro B., Schwartz M., Sherman M., Gores G. (2016). Hepatocellular carcinoma. Nat. Rev. Dis. Prim..

[2] Llovet J.M., Ricci S., Mazzaferro V., Hilgard P., Gane E., Blanc J.-F., De Oliveira A.C., Santoro A., Raoul J.-L., Forner A. (2008). Sorafenib in Advanced Hepatocellular Carcinoma. N. Engl. J. Med..

[3] Kudo M., Finn R.S., Qin S., Han K.-H., Ikeda K., Piscaglia F., Baron A., Park J.-W., Han G., Jassem J. (2018). Lenvatinib versus sorafenib in first-line treatment of patients with unresectable hepatocellular carcinoma: A randomised phase 3 non-inferiority trial. Lancet.

[4] Bruix J., Qin S., Merle P., Granito A., Huang Y.-H., Bodoky G., Pracht M., Yokosuka O., Rosmorduc O., Breder V. (2017). Regorafenib for patients with hepatocellular carcinoma who progressed on sorafenib treatment (RESORCE): A randomised, double-blind, placebo-controlled, phase 3 trial. Lancet.

[5] Zhu A.X., Kang Y.-K., Yen C.-J., Finn R.S., Galle P.R., Llovet J.M., Assenat E., Brandi G., Pracht M., Lim H.Y. (2019). Ramucirumab after sorafenib in patients with advanced hepatocellular carcinoma and increased α-fetoprotein concentrations (REACH-2): A randomised, double-blind, placebo-controlled, phase 3 trial. Lancet Oncol..

[6] Cremolini C., Loupakis F., Antoniotti C., Lonardi S., Masi G., Salvatore L., Cortesi E., Tomasello G., Spadi R., Zaniboni A. (2015). Early tumor shrinkage and depth of response predict long-term outcome in metastatic colorectal cancer patients treated with first-line chemotherapy plus bevacizumab: Results from phase III TRIBE trial by the Gruppo Oncologico del Nord Ovest. Ann. Oncol..

[7] Heinemann V., Modest D., Von Weikersthal L.F., Decker T., Kiani A., Vehling-Kaiser U., Al-Batran S.-E., Heintges T., Lerchenmüller C., Kahl C. (2014). Independent Radiological Evaluation of Objective Response Early Tumor Shrinkage, and Depth of Response in FIRE-3 (AIO KRK-0306). Ann. Oncol..

[8] Heinemann V., Stintzing S., Modest D.P., Gießen-Jung C., Michl M., Mansmann U. (2015). Early tumour shrinkage (ETS) and depth of response (DpR) in the treatment of patients with metastatic colorectal cancer (mCRC). Eur. J. Cancer.

[9] Vivaldi C., Fornaro L., Cappelli C., Pecora I., Catanese S., Salani F., Insilla A.C., Kauffmann E.F., Donati F., Pasquini G. (2019). Early Tumor Shrinkage and Depth of Response Evaluation in Metastatic Pancreatic Cancer Treated with First Line Chemotherapy: An Observational Retrospective Cohort Study. Cancers.

[10] Miyake H., Miyazaki A., Imai S., Harada K.-I., Fujisawa M. (2015). Early Tumor Shrinkage Under Treatment with First-line Tyrosine Kinase Inhibitors as a Predictor of Overall Survival in Patients with Metastatic Renal Cell Carcinoma: A Retrospective Multi-Institutional Study in Japan. Target. Oncol..

[11] Kawachi H., Fujimoto D., Morimoto T., Hosoya K., Sato Y., Kogo M., Nagata K., Nakagawa A., Tachikawa R., Tomii K. (2019). Early depth of tumor shrinkage and treatment outcomes in non-small cell lung cancer treated using Nivolumab. Investig. New Drugs.

[12] Therasse P., Arbuck S.G., Eisenhauer E.A., Wanders J., Kaplan R.S., Rubinstein L., Verweij J., Van Glabbeke M., Van Oosterom A.T., Christian M.C. (2000). New Guidelines to Evaluate the Response to Treatment in Solid Tumors. J. Natl. Cancer Inst..

[13] Lencioni R., Llovet J.M. (2010). Modified RECIST (mRECIST) Assessment for Hepatocellular Carcinoma. Semin. Liver Dis..

[14] Kudo M., Finn R.S., Qin S., Han K.-H., Ikeda K., Cheng A.-L., Piscaglia F., Ueshima K., Aikata H., Vogel A. (2019). Analysis of survival and objective response (OR) in patients with hepatocellular carcinoma in a phase III study of lenvatinib (REFLECT). J. Clin. Oncol..

[15] Takahashi A., Moriguchi M., Seko Y., Ishikawa H., Yo T., Kimura H., Fujii H., Shima T., Mitsumoto Y., Ishiba H. (2019). Impact of Relative Dose Intensity of Early-phase Lenvatinib Treatment on Therapeutic Response in Hepatocellular Carcinoma. Anticancer. Res..

[16] Bruix J., Cheng A.-L., Meinhardt G., Nakajima K., De Sanctis Y., Llovet J.M. (2017). Prognostic factors and predictors of sorafenib benefit in patients with hepatocellular carcinoma: Analysis of two phase III studies. J. Hepatol..

[17] Llovet J.M., Pena C.E.A., Lathia C.D., Shan M., Meinhardt G., Bruix J. (2012). Plasma Biomarkers as Predictors of Outcome in Patients with Advanced Hepatocellular Carcinoma. Clin. Cancer Res..

[18] Yamashita T., Kudo M., Ikeda K., Izumi N., Tateishi R., Ikeda M., Aikata H., Kawaguchi Y., Wada Y., Numata K. (2019). REFLECT—A phase 3 trial comparing efficacy and safety of lenvatinib to sorafenib for the treatment of unresectable hepatocellular carcinoma: An analysis of Japanese subset. J. Gastroenterol..

[19] Eso Y., Nakano S., Mishima M., Arasawa S., Iguchi E., Nakamura F., Takeda H., Takai A., Takahashi K., Taura K. (2019). Dose Intensity/Body Surface Area Ratio is a Novel Marker Useful for Predicting Response to Lenvatinib against Hepatocellular Carcinoma. Cancers.

[20] Hatanaka T., Kakizaki S., Nagashima T., Namikawa M., Tojima H., Shimada Y., Takizawa D., Naganuma A., Arai H., Sato K. (2019). Analyses of objective response rate, progression-free survival, and adverse events in hepatocellular carcinoma patients treated with lenvatinib: A multicenter retrospective study. Hepatol. Res..

[21] Hiraoka A., Kumada T., Atsukawa M., Hirooka M., Tsuji K., Ishikawa T., Takaguchi K., Kariyama K., Itobayashi E., Tajiri K. (2019). Prognostic factor of lenvatinib for unresectable hepatocellular carcinoma in real-world conditions-Multicenter analysis. Cancer Med..

[22] Mazard T., Boonsirikamchai P., Overman M., Asran M.A., Choi H., Herron D., Eng C., Maru D., Ychou M., Vauthey J.-N. (2017). Comparison of early radiological predictors of outcome in patients with colorectal cancer with unresectable hepatic metastases treated with bevacizumab. Gut.

[23] Terashima T., Yamashita T., Takata N., Nakagawa H., Toyama T., Arai K., Kitamura K., Yamashita T., Sakai Y., Mizukoshi E. (2015). Post-progression survival and progression-free survival in patients with advanced hepatocellular carcinoma treated by sorafenib. Hepatol. Res..

[24] Ogasawara S., Chiba T., Ooka Y., Suzuki E., Kanogawa N., Saito T., Motoyama T., Tawada A., Kanai F., Yokosuka O. (2016). Post-progression survival in patients with advanced hepatocellular carcinoma resistant to sorafenib. Investig. New Drugs.

[25] Kuzuya T., Ishigami M., Ishizu Y., Honda T., Hayashi K., Ishikawa T., Nakano I., Hirooka Y., Goto H. (2018). Prognostic Factors Associated with Postprogression Survival in Advanced Hepatocellular Carcinoma Patients Treated with Sorafenib Not Eligible for Second-Line Regorafenib Treatment. Oncology.

[26] Lencioni R., Montal R., Torres F., Park J.-W., Decaens T., Raoul J.-L., Kudo M., Chang C., Ríos J., Boige V. (2017). Objective response by mRECIST as a predictor and potential surrogate end-point of overall survival in advanced HCC. J. Hepatol..

[27] Meyer T., Palmer D.H., Cheng A.-L., Hocke J., Loembé A., Yen C. (2017). mRECIST to predict survival in advanced hepatocellular carcinoma: Analysis of two randomised phase II trials comparing nintedanib vs sorafenib. Liver Int..

[28] Takeda H., Nishijima N., Nasu A., Komekado H., Kita R., Kimura T., Kudo M., Osaki Y. (2019). Long-term antitumor effect of lenvatinib on unresectable hepatocellular carcinoma with portal vein invasion. Hepatol. Res..

[29] Kuzuya T., Ishigami M., Ito T., Ishizu Y., Honda T., Ishikawa T., Fujishiro M. (2019). Favorable radiological antitumor response at 2 weeks after starting lenvatinib for patients with advanced hepatocellular carcinoma. Hepatol. Res..

[30] Ueshima K., Nishida N., Hagiwara S., Aoki T., Minami T., Chishina H., Takita M., Minami Y., Ida H., Takenaka M. (2019). Impact of Baseline ALBI Grade on the Outcomes of Hepatocellular Carcinoma Patients Treated with Lenvatinib: A Multicenter Study. Cancers.

[31] Kodama K., Kawaoka T., Namba M., Uchikawa S., Ohya K., Morio K., Nakahara T., Murakami E., Yamauchi M., Hiramatsu A. (2019). Correlation between Early Tumor Marker Response and Imaging Response in Patients with Advanced Hepatocellular Carcinoma Treated with Lenvatinib. Oncology.

[32] Sasaki R., Fukushima M., Haraguchi M., Miuma S., Miyaaki H., Hidaka M., Eguchi S., Matsuo S., Tajima K., Matsuzaki T. (2019). Response to Lenvatinib Is Associated with Optimal RelativeDose Intensity in Hepatocellular Carcinoma: Experience in Clinical Settings. Cancers.

[33] Arizumi T., Ueshima K., Chishina H., Kono M., Takita M., Kitai S., Inoue T., Yada N., Hagiwara S., Minami Y. (2014). Duration of Stable Disease Is Associated with Overall Survival in Patients with Advanced Hepatocellular Carcinoma Treated with Sorafenib. Dig. Dis..

[34] Hiraoka A., Kumada T., Tsuji K., Takaguchi K., Itobayashi E., Kariyama K., Ochi H., Tajiri K., Hirooka M., Shimada N. (2018). Validation of Modified ALBI Grade for More Detailed Assessment of Hepatic Function in Hepatocellular Carcinoma Patients: A Multicenter Analysis. Liver Cancer.

[35] Marrero J.A., Kulik L.M., Sirlin C.B., Zhu A.X., Finn R.S., Abecassis M., Roberts L.R., Heimbach J.K. (2018). Diagnosis, Staging, and Management of Hepatocellular Carcinoma: 2018 Practice Guidance by the American Association for the Study of Liver Diseases. Hepatology.

[36] Pencina M.J., D’Agostino R.B. (2004). OverallC as a measure of discrimination in survival analysis: Model specific population value and confidence interval estimation. Stat. Med..

