# Supplementary material for: Early Tumor Shrinkage as a Predictive Factor for Outcomes in Hepatocellular Carcinoma Patients Treated with Lenvatinib: A Multicenter Analysis
Source: Cancers (Basel). 2020 Mar 23;12(3):754. doi: 10.3390/cancers12030754 (PMC7140019; doi:10.3390/cancers12030754)

Figure S2. Subgroup analyses for overall survival. ETS, early tumor shrinkage; CPS, Child-Pugh score; MVI, macrovasucular invasion.

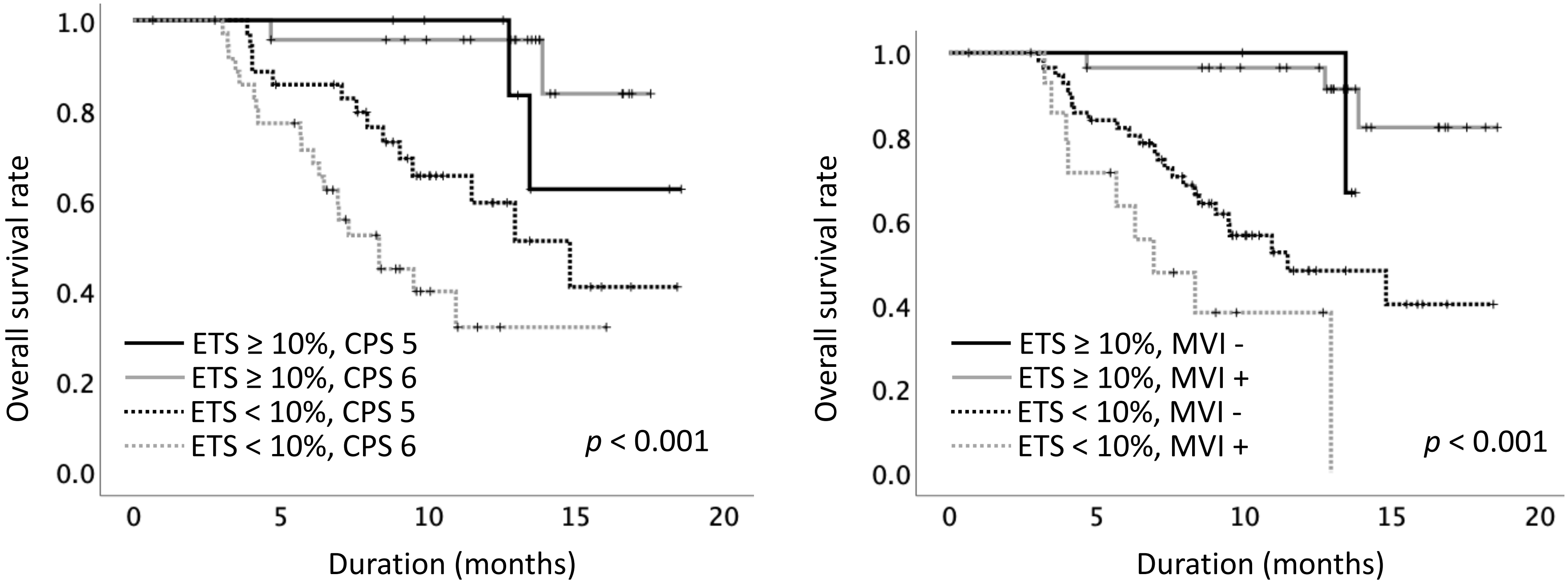

Supplement: Supplementary file 1 [file cancers-12-00754-s001.zip › Figure S2 cancers.pdf]
