# Supplementary material for: Early Tumor Shrinkage as a Predictive Factor for Outcomes in Hepatocellular Carcinoma Patients Treated with Lenvatinib: A Multicenter Analysis
Source: Cancers (Basel). 2020 Mar 23;12(3):754. doi: 10.3390/cancers12030754 (PMC7140019; doi:10.3390/cancers12030754)

Figure S1.

Receiver operating characteristic curves of the association of long-term response (PFS ≥ 5.0 months) with AFP ratio and RDI at 8 weeks. PFS, progression-free survival; AFP, α-fetoprotein; RDI, relative dose intensity; AUC, area under the curve

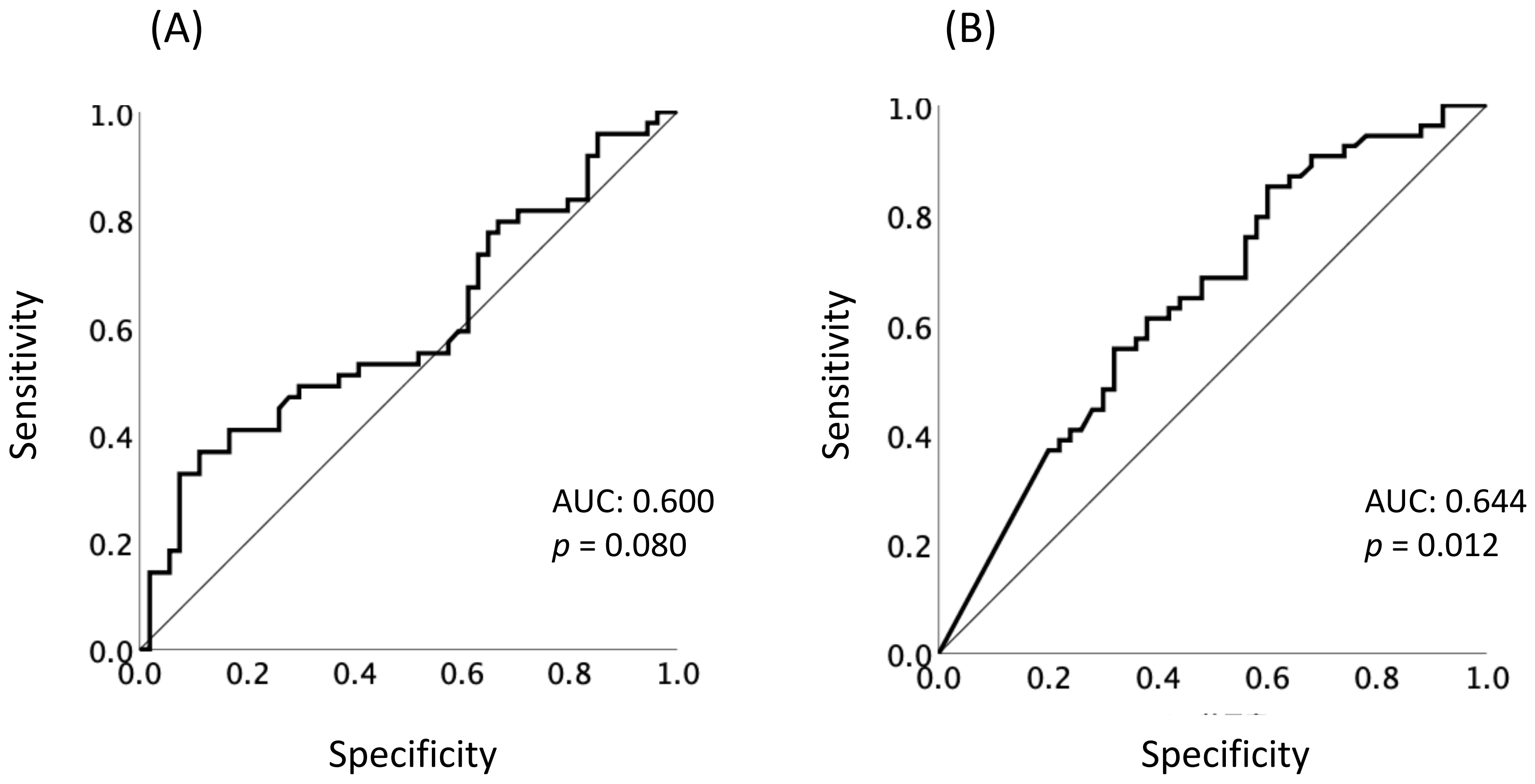

Supplement: Supplementary file 1 [file cancers-12-00754-s001.zip › Figure S1 cancers.pdf]
